# Indoor Localization Based on Infrared Angle of Arrival Sensor Network

**DOI:** 10.3390/s20216278

**Published:** 2020-11-04

**Authors:** Damir Arbula, Sandi Ljubic

**Affiliations:** University of Rijeka, Faculty of Engineering, 51000 Rijeka, Croatia; damir.arbula@riteh.hr

**Keywords:** infrared sensor, angle of arrival, indoor localization, wireless sensor networks, navigation

## Abstract

Accurate, inexpensive, and reliable real-time indoor localization holds the key to the full potential of the context-aware applications and location-based Internet of Things (IoT) services. State-of-the-art indoor localization systems are coping with the complex non-line-of-sight (NLOS) signal propagation which hinders the use of proven multiangulation and multilateration methods, as well as with prohibitive installation costs, computational demands, and energy requirements. In this paper, we present a novel sensor utilizing low-range infrared (IR) signal in the line-of-sight (LOS) context providing high precision angle-of-arrival (AoA) estimation. The proposed sensor is used in the pragmatic solution to the localization problem that avoids NLOS propagation issues by exploiting the powerful concept of the wireless sensor network (WSN). To demonstrate the proposed solution, we applied it in the challenging context of the supermarket cart navigation. In this specific use case, a proof-of-concept navigation system was implemented with the following components: IR-AoA sensor prototype and the corresponding WSN used for cart localization, server-side application programming interface (API), and client application suite consisting of smartphone and smartwatch applications. The localization performance of the proposed solution was assessed in, altogether, four evaluation procedures, including both empirical and simulation settings. The evaluation outcomes are ranging from centimeter-level accuracy achieved in static-1D context up to 1 m mean localization error obtained for a mobile cart moving at 140 cm/s in a 2D setup. These results show that, for the supermarket context, appropriate localization accuracy can be achieved, along with the real-time navigation support, using readily available IR technology with inexpensive hardware components.

## 1. Introduction

In recent years, we have been witnessing a rapid increase in the availability of commercial indoor localization solutions. This is not a surprise as smartphone users are already accustomed to outdoor location-based services. Precise indoor localization is the only significant technological obstacle to extend these services to the area where many users spend most of their time. Indoor localization is, therefore, the Holy Grail problem in ubiquitous computing, context-aware applications, and, specifically, location-based Internet of Things (IoT) services.

The outdoor localization problem is solved by Global Positioning System (GPS), a satellite-based navigation system consisting of a network of 24 satellites placed into orbit. The basis of the outdoor localization method is the combination of the line-of-sight (LOS) radio propagation from the satellite transmitter to the receiver and the fact that it can be predicted and even real-time calibrated using information from the referent stations on the ground. Distance from the satellites with known locations can be precisely estimated based on the time it takes for the signal to reach the receiver; therefore, the receiver can be positioned using the multilateration method.

Unlike in the open spaces, signal propagation indoors is affected by complex interactions with a large number of fixed and moving obstacles through reflection, refraction, and scattering. Hence, it is very difficult or even not possible to predict the signal path and to correlate distance from the receiver with the propagation time, signal strength, or any other signal parameter. As noted in the following subsection, where we give an overview of the commercial systems and research solutions to the indoor localization problem, non-line-of-sight (NLOS) and multipath propagation is the main problem that various authors try to solve or even avoid. For example, Belloni et al. [1] target their application to obstacle-free open indoor spaces since the angle-of-arrival (AoA) method is very sensitive to multipath propagation, resulting in poor localization accuracy. On the other hand, the LOS signal propagation is characterized by the fact that the intensity of the LOS component of the signal is significantly higher than other components. Thus, the signal propagation path between transmitter and receiver can be modeled with a straight line, allowing for precise and accurate transmitter location estimation.

Indoor positioning systems (IPS) face an interesting technical challenge due to the wide variety of promising sensor technologies that can be applied, each one with different pros and cons. Brena et al. [2] provided a helpful systematization of the respective field, by introducing a comprehensive review of the literature that involves the technological perspective of IPS evolution, a classification scheme for different technological approaches, and a presentation of the existing research trends. In the related survey, authors concluded that “there is not yet an overall satisfying solution for the IPS problem”, and, while addressing the specific problem of locating merchandise in retail stores, they argued that “not a single technology or combination of technologies is both feasible and satisfying”. Shang et al. [3] have also presented a detailed survey in this field, however, they focused on a review of the improvement schemes for indoor mobile location estimation. Among many methods and techniques for enhancing location estimation, they analyzed the possibility of fusing spatial context. Namely, they tackled graph-based motion models of an indoor space, an instance of which we utilized in our research.

The indoor localization field is broad and there are many different solutions but their performance is very difficult to evaluate without the proper context. Therefore, in the following overview, we specifically focus on systems that target or can be used in our showcase application: supermarket aisle-level localization, which itself has been given a lot of attention, specifically cart localization within high shelves surrounded corridors.

### 1.1. Related Work: Indoor Localization Methods and Solutions

In this subsection, we present current state-of-the-art indoor localization systems, both in the research and development phase, as well as those that are already commercially available. Presented systems are roughly divided into two groups, based on the type of signal used, and one additional group consisting of infrastructure-free systems that require no signal (Table 1).

Infrastructure based methods are using either radio frequency (RF) signals or modulated visible light sources to be able to estimate the position and, as such, require specific infrastructure to be installed on-site. Infrastructure, in this sense, consists of highly available WiFi access points (AP) or specialized equipment that has to be installed with specific localization intent, such as radio-frequency identification (RFID) scanners, Bluetooth Low Energy (BLE) beacons, Ultra-wideband (UWB) beacons, or modulated light sources.

#### 1.1.1. Systems Using RF Signal

WiFi is the dominating wireless technology for indoor data transfer and, as such, the WiFi signal is the ideal candidate to exploit for localization purposes. WiFi localization systems are predominantly using Received Signal Strength (RSS) as the principal method for the estimation of the distance between WiFi AP and mobile nodes. Standard indoor NLOS multipath propagation decreases the correlation between distance and RSS to the point where, using estimated distances, only basic proximity-based localization is possible. On the other hand, short range and the abundance of different available WiFi APs are enabling a pattern matching method or fingerprinting to become the de-facto standard localization technique for indoor location-based services on consumer devices [4]. However, this method requires a site survey fingerprint in advance and localization performance is highly sensitive to changes in the environment, i.e., in a supermarket with moving people. The most common algorithm utilized for WiFi fingerprinting is weighted K-nearest neighbors (WKNN), which calculates K-nearest neighboring points to a mobile user. Typical problems associated with the WKNN involve a difference in observed AP sets during offline and online stages, and a possibility for some of the K neighbors to be physically far from the user. Enhancements of the default WKNN have thus been proposed, that change the number of considered neighbors dynamically—either by using RSS-based filtering [5], or a more sophisticated clustering algorithm [6]. Issues and requirements of the WiFi-based localization systems are furthermore alleviated by the incorporation of inertial motion unit (IMU) data readily available on modern smartphone devices and by using the Simultaneous Localization and Mapping (SLAM) technique. Systems based on this technique, such as Apple WiFiSLAM [7], achieve localization accuracy around 2 m. Yang and Shao [8] obtained even more promising results by using multiple antennas on WiFi APs and the combination of distance and AoA estimation along with the capability of filtering NLOS measurements. The authors report localization error from 2.2 m up to 0.5 m by using one or several WiFi access points, respectively. WiDeo system, introduced in Reference [9], represents one of the most encouraging efforts in providing WiFi-based indoor motion tracking. It utilizes specially developed WiFi AP with antenna array, as well as backscatter analysis, i.e., composite reflected signal examination wherein the amplitude, time-of-flight (ToF), and AoA parameters are all estimated. The WiDeo thus provides a possibility to trace subject motions without the need for any accompanying device, with reported median localization accuracy of 0.8 m and motion tracking accuracy of 7 cm. Since WiDeo’s accuracy is in line with the localization accuracy of the solution proposed in this paper, an appropriate comparison is given in the Discussion section, highlighting the pros-and-cons for using the related systems in the target supermarket environment.

Systems using Bluetooth Low Energy (BLE) technology are commercialized under different brand names, such as iBeacon by Apple or Eddystone by Google. They consist of a number of beacons with known positions publishing their ID and mobile nodes that are localized through estimated distances from beacons using the multilateration method. This method is somewhat similar to the WiFi RSS method, but a low range warrants smaller cells and lower distance estimation errors. Just like with WiFi, the advantage of this method is its availability on all present-day smartphones, while, at the same time, deployed devices are cheaper, smaller, more portable, and energy-efficient. Nevertheless, it is hard to achieve sub-meter precision, i.e., Faragher and Harle [10] report tracking accuracies of <2.6 m in 95% of the time with a density of one beacon in 30 m^2^. Currently, these systems are mostly used for proximity-based localization and point-of-interest services.

Furthermore, BLE and WiFi can be combined. Kriz et al. [11] report sub-meter localization error median in a 52 × 43 m office building equipped with 4 WiFi access points and 17 BLE beacons. The downside of their method is a relatively long scan and measure delay taking from 6 to 10 s to reach stated localization accuracy. There are many commercial systems present relying on WiFi and BLE, such as AisleLabs, and those that are combining BLE, WiFi, and SLAM methods, such as indoo.rs. The latter combination has reported accuracy from 2 to 5 m, depending on the density and placement of beacons.

Ultra-wideband (UWB) is an RF technology for a short-range, high bandwidth communication with a high temporal resolution, resulting in centimeter-level accuracy [12]. Localization systems based on UWB are mostly using time-of-arrival (ToA) and time-difference-of-arrival (TDoA) of RF signals to estimate the distance between the transmitter and the receiver. To be able to use ToA methods they need to perform precise time synchronization of anchor nodes, somewhat similar to GPS satellites. Although there are techniques that mitigate this synchronization challenge [13], this requirement further complicates the overall system. Commercial UWB systems, such as Sewio, utilize many anchor nodes in the LOS range of mobile nodes they are tracking. Although the accuracy of UWB systems is very competitive, the limited range of the anchor nodes in the supermarket configuration, along with their specialized hardware design, and consequently high price, results in prohibitive installation costs.

Radio-frequency identification (RFID) is fairly mature and available technology mainly used for object tagging and identification, but there are also many examples of using RFID in indoor localization scenarios. One of the first usages was the LANDMARC system [14] that consisted of a small number of RFID readers with a high range and a large number of active RFID tags divided into two sets: landmark tags with known locations and mobile tags with unknown locations. Landmark tags were used for continuous calibration providing partial resistance to changes in the environment, thus enabling more accurate mobile tag location estimation. Reported accuracy is from 1 to 2 m, but authors did note several important issues, such as long scan time (7.5 s interval between readings) and inconsistent emitting signal strengths of RFID tags. Ryoo and Das [15] utilized RFID to enable supermarket cart localization. They report a median of localization error limited to 5 cm with a 90-percentile error of 15 cm. This method is using carts equipped with passive RFID tags, while RFID readers are installed directly above the aisle on a fixed height 2 m above the cart. The localization algorithm is based on a distance estimation between the reader and the tag. Distance is estimated using Δϕ/Δf slope obtained from phase response measurements through different interrogation channels. The problematic aspect of this method is long measurement time, around 400 ms for each channel. Since there is a minimum of 5 channels, it adds up to 2 s during which the cart has to remain stationary in order to estimate distance and location. Other drawbacks of this system include the high cost of multiple RFID readers, each with multiple antenna setup and appropriate cabling.

#### 1.1.2. Systems Using Light Sources

Another signal source that can be exploited for localization purposes is light, either infrared or visible. With light-emitting diode (LED) technology becoming the new standard in ambient illumination there are numerous possibilities to harness its properties, such as Visible Light Communication (VLC) and Visible Light Positioning (VLP) [16]. The basic principle of VLP operation is that each light source serves as a beacon whose modulated radiation can be captured by a light sensor, usually a front-facing smartphone camera. Radiation from each light source is modulated (e.g., by fixed frequency or by transmitting Manchester-encoded data), and can be uniquely identified by a mobile node. The identification of multiple light sources in an image allows the positioning of a smartphone using AoA. There are many examples of VLC and VLP systems both in research and development and in the commercial phase. Kuo et al. [17] present the Luxapose system which, in a laboratory environment, achieves decimeter-level localization accuracy using a high resolution 33 MP smartphone camera and 5 LED beacons. On the other hand, Qiu et al. [18] are using simple and inexpensive external light sensors in a 4.7 m × 8.6 m indoor environment with 12 modulated LEDs, attaining sub-meter precision. Their approach requires a data collection phase similar to the WiFi fingerprinting method. Among commercially available systems, we can highlight those from companies, like ByteLight, GE Lighting, and Philips [19]. Typical drawbacks of VLP systems are considerable computational requirements for real-time image processing and location estimation and high energy demands, as well as high initial installation cost.

Infrared (IR) communication technology is widely adopted, inexpensive and readily available. IR signals are used in many different applications ranging from consumer remote controls to data transfer (IrDA). One of the first indoor localization systems Active Badge [20] was using IR signals. This system was intended for personnel tracking using a set of tags each emitting IR signal with a unique code every 15 s. Signals are picked up by Badge Sensors installed at various rooms inside the building providing room-level accuracy. Badge Sensors were powered and connected to a network using a special 4 wire system using telephone twisted-pairs cable and RS232 data-transfer format.

In more recent research [21], IR beacons are detected using a low-resolution CCD camera fitted with an IR filter on a mobile robot, a method similar to the already mentioned VLP solution [17]. Although the setup seems simple, and only a small number of LEDs in a field-of-view (FoV) is required to achieve decimeter-level precision, the problem is that the beacon signal is not identifiable; thus, their positions are hardcoded. To be able to identify beacons, a large number of modulated LED sources needs to be installed and powered full time. This requires an adequate energy source, either through separate cabling or battery, both options being rather expensive.

The problem of multipath (MP) propagation is the most prominent among the indoor positioning systems based on optical signals. Namely, the receiver in such systems usually senses the line of sight component of the signal, as well as other MP components, due to light reflections and refractions in the indoor environment. Since the received signal components can vary in power strength and phase, the localization accuracy of the underlying system can be significantly reduced.

A model of IR signal reflections on any kind of surface material is proposed in Reference [22] that can be applied to characterize the multipath behavior of optical signals in applications, such as indoor positioning and VLC communications. The respective model is derived according to the experimental measurements on three different materials (terrazzo, foam board, and plasterboard). In Reference [23], authors propose a model to determine the multipath effect in indoor environments when the shape and characteristics of the environment (e.g., reflection features of the materials) are known a priori. The related model can be applied for indoor positioning, irrespectively, of both the underlying system and the utilized measurement type (e.g., RSS, phase of arrival (PoA), differential phase of arrival (DPoA)). For example, when analyzing the MP effect in AoA-based systems, wherein the signal phase information is not relevant, it is necessary to know the signal strength reaching the detector from each element in the environment after a certain number of rebounds. The mentioned model comes with an algorithm that calculates the signal strength in the MP scenario. In recent research [24], a Position Sensitive Device (PSD) sensor was used for experimental testing of MP effects in IR-based indoor positioning. The positioning has been calculated using AoA and PoA techniques, and the errors caused by the MP have been analyzed. The obtained results showed that the MP effects for AoA, unlike for PoA, have little impact on the indoor positioning accuracy.

#### 1.1.3. Infrastructure-Free Systems

Unlike systems that are based on specific installed infrastructure providing RF or light signals and allowing estimation of distance, angle, and consequently position, some systems demand no specific equipment to estimate indoor location.

The first system of that kind uses the fact that the Earth’s magnetic field is distorted by structural steel elements in a building and that this distorted field has a certain temporally stable signature that can be mapped. Related methods are somewhat similar to the WiFi fingerprinting, the key difference being that the Earth’s magnetic field is stable and undisturbed even by large moving metal objects (i.e., elevator cabin) on distances above 1 m from the magnetic sensor. Chung et al. [25] report accuracy within 1.64 m for 90% of the time using a simple RMS-based nearest neighbor searching algorithm for the localization. On the other hand, they also note that the chance of error increases with the size of the fingerprint map and propose a hybrid solution with WiFi fingerprinting that can complement repeating magnetic signatures and set upper bound on localization error for larger maps. This method is further investigated by Shu et al. [26], along with the more sophisticated augmented particle filter (APF) localization algorithm and IMU-based tracking used to help in the proper timing of the magnetic field measurements. The interesting fact in the context of this paper is that the authors describe the supermarket environment as the most challenging one (others being office building and underground parking garage). Their experimental results verify that description since they report that their system achieves 90 percentile localization accuracy of 8 m in the supermarket environment using the magnetic field alone. Finally, it is worth noticing that although the infrastructure is not required, this method requires mapping the magnetic field which can take significant effort and time. Representative commercial implementation of the magnetic field sensing is one by the brand IndoorAtlas.

Another innovative system that requires no infrastructure is Google Tango Project. Three key features of the Tango Project system are (1) motion tracking, (2) area learning, and (3) depth perception. Tango can be used both to map indoor spaces and to estimate location within by using a standard gyroscope, accelerometers along with the wide-angle camera and depth techniques, such as Structured Light, Time of Flight, and Stereo Vision. The project is still in its research and development phase, and Tango-enabled devices are becoming available on the market only recently. Tango-enabled devices can be used in indoor localization and navigation context, and, currently, besides the unavailability of the hardware, the main obstacle is the power required for computation.

Finally, it is worth mentioning the Monocular localization system [27] that can be used as a complement to Google Tango. This system uses real-time video camera-based optical character recognition (OCR) and building floor plan with a mapping of prominent signs locations, such as store logos above entrances. Using this information, it is possible to estimate location relative to a detected visual cue.

Based on the research review presented above, a comparison of indoor localization systems’ main characteristics is summarized in Table 2. Along with the characteristics of different localization methods (typical accuracy, installation costs estimate, energy consumptions, and main drawbacks), we highlight the representative commercial solutions that can be considered as readily available for applying in the supermarket context. By outlining the commercial examples, we point out the fact that some of the largest (and the most influential) companies, such as Apple, Philips, and Google, recognize the importance of indoor localization, and actively contribute in the respective field.

### 1.2. The Overview of the Proposed Solution

In this paper, we present a novel infrared (IR) sensor and AoA estimation algorithm relying on low range LOS signal propagation. The sensor is furthermore applied in a novel localization method based on tracking mobile IR transmitters. In order to provide LOS signal sensing throughout the environment and gather measurements from mobile nodes, we exploit the powerful concept of the wireless sensor network (WSN). We believe that this combination has the potential to overcome some of the issues within current state-of-the-art indoor localization systems.

Although the proposed method can be utilized in many different applications, in this paper, we tackle the specific aisle-level cart navigation use case. The environment in this use case is characterized by many narrow corridors, high or moving obstacles, such as shelves or customers. Many current systems fall short in this kind of environment, specifically because of the unpredictable and changing signal propagation. In this context, our showcase system is using WSN nodes which are equipped with IR AoA sensors and distributed above the aisles. WSN measures a signal from the infrared transmitters installed on the carts and delivers those measurements to the localization server, thus enabling real-time cart localization. The key advantages of this system are inexpensive installation and maintenance, and competitive localization precision as demonstrated in conducted experiments.

The related research efforts most often focus exclusively on the design of a specific sensor with an attempt to enhance indoor localization accuracy but without further utilization within a system that would assist the end-user to navigate in the target indoor environment. In other words, the related work often lacks the well-rounded solution built upon the underlying localization technology. In this sense, our contributions are based on the development of all modules required for indoor navigation and their successful integration into the proof-of-concept system. The system targets the supermarket navigation context and involves the following:

- Novel IR AoA sensor, made of inexpensive off-the-shelf components, enabling AoA estimation with an error around 1°,
- Wireless sensor network, based on the proposed IR AoA sensor, which provides infrastructural support for real-time navigation,
- Localization strategy/method/algorithm, utilizing the proposed WSN and a spatial context (aisle graph), with suitable localization accuracy,
- Supermarket navigation model based on shelves graph and aisles graph,
- Server, API, and client applications suite, demonstrating both the features and the look-and-feel of the proposed system.

## 2. Materials and Methods

### 2.1. Angle-of-Arrival Sensor

As with every conventional outdoor navigation system, the integral component of the indoor navigation system is the one used for mobile node localization. The proposed method consists of measuring the strength of the IR signal on the IR phototransistors placed on a specifically constructed sensor and the estimation of the angle-of-arrival of the IR signal from the measurement data. This localization method achieves high accuracy with simple low-cost hardware, while requiring LOS between the IR transmitter and IR sensor. Therefore, the key technical properties are novel sensor design and angle-of-arrival estimation algorithm.

In our research, we opted for IR-based technology, with two main goals in mind: (1) to propose a LOS-based sensor design that would be inexpensive to produce—using cheap off-the-shelf components, and (2) to develop the proof-of-concept solution, utilizing the network of such sensors, that would be fittingly accurate in the target (supermarket) context.

When it comes to the already available sensors that provide AoA measurements, we can outline devices used in the Cricket Compass System [28] working with ultrasound signals, different antenna array systems [29], and rotating laser systems [30]. Furthermore, a PSD (Position Sensitive Device) sensor has been successfully utilized for indoor positioning using AoA techniques, with the obtained localization error below 1 cm [31]. QADA (Quadrant Photodiode Angular Diversity Aperture) sensor also showed to be a part of the promising angle-based localization apparatus, as it was used in an IR indoor positioning system that provides the absolute error of 0.9° in the estimation of the polar angle, and 12 cm of absolute localization error [32].

However, all these mentioned solutions are neither simple nor low-priced nor fully adequate for straightforward installation in the supermarket venue. For example, some of them require appropriate cabling, which we wanted to avoid from the very beginning. Regarding the sensor costs, we can outline PSD and QADA devices that were used in the abovementioned research (Hamamatsu S5991-01 PSD, and QADA receiver QP50-6-18u-TO8) and which hold a price level of USD 180–200 and USD 120, respectively. Following our main idea, we opted for a novel design of a much more affordable AoA sensor.

#### 2.1.1. Design

The inspiration for the creation of a new type of sensor was drawn from the research by Song et al. [33], in which authors introduce a new type of digital camera. The camera has a size of 1 cm in diameter and contains a total of 180 micro-lens oriented in different directions [34].

The key idea of the proposed design is to utilize an array of IR phototransistors placed on the circular rim and directed outwards to detect the angle of arrival (AoA) of the incoming infrared signal. AoA could be estimated using measured data and known specific radiant sensitivity of IR phototransistors.

Initial advantages of this method are the usage of small and inexpensive off-the-shelf components, such as IR phototransistors, along with the readily available dedicated ATtiny45 microcontroller and the 16-channel multiplexer, as well as the fact that the principle of the operation of the hardware part is rather simple. The sensor is controlled via a one-wire protocol that is used to select the appropriate channel on the multiplexer, allowing the measurement of selected IR phototransistor output by the host node AD converter.

**Figure 1:**
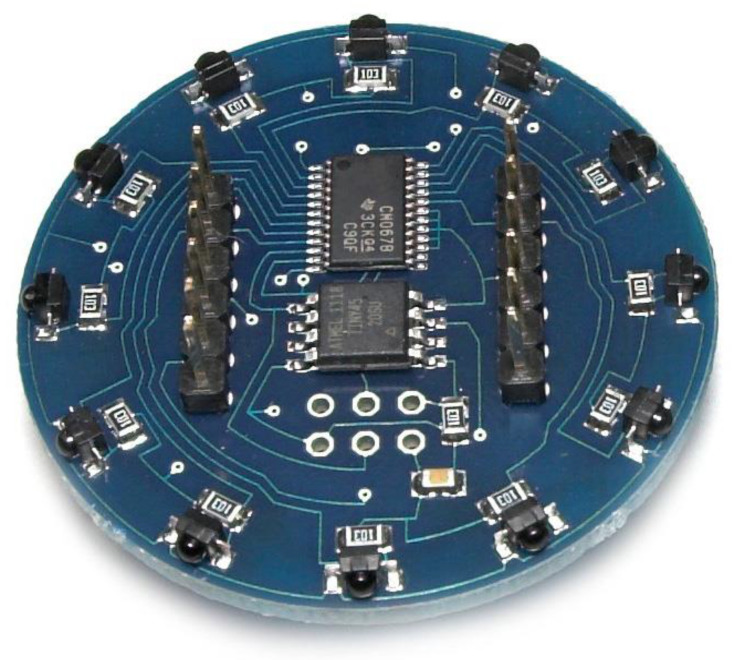
Infrared (IR) angle-of-arrival (AoA) sensor prototype.

The first-generation prototype was our preliminary design of the novel target IR AoA sensor, which showed to be only a “debugging” step in the process of building the final, i.e., the second-generation prototype. Namely, the first-generation prototype used a simple design with through-hole components and was able to estimate AoA with an average error of around 10°. We found this error to be quite large, so we did not consider the related prototype for further work on the localization system. Instead, we tackled the unfitting size and imprecise positioning of IR phototransistors (in the first-generation prototype) by introducing surface mounted (SMT) components and pick-and-place automated assembly. By doing so, we developed the second-generation prototype (shown in Figure 1) with the lower AoA estimation error. We were able to further reduce this error, up to 1°, by utilizing a specific calibration procedure and the corresponding estimation algorithm (described in the following subsections). The second-generation prototype was thus used in our WSN-based localization solution and related experiments.

#### 2.1.2. Calibration

**Figure 2:**
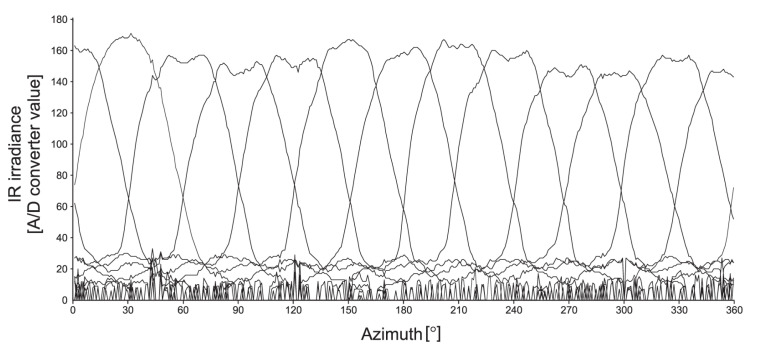
AoA phototransistors’ IR irradiance measurements with varying angle of arrival of the incoming IR signal. Each curve corresponds to one of 12 phototransistors on the AoA sensor. Maximal values for each phototransistor are achieved when the transmitter is positioned near the phototransistor axis, i.e., directly in front of the phototransistor. IR irradiance is measured as a voltage drop on resistors serially connected to phototransistors. The phototransistor collector current and the corresponding voltage drop are proportional to the measured irradiance. The voltage is displayed as a 10-bit A/D converter readout.

The main source of estimation error proved to be the relative radiant sensitivity of each SMT IR phototransistor, which is unique (Figure 2) and significantly different from the ideal characteristic specified in the datasheet [35]. This fact presented an issue since radiant sensitivity characteristic is the principal parameter for enabling AoA estimation.

**Figure 3:**
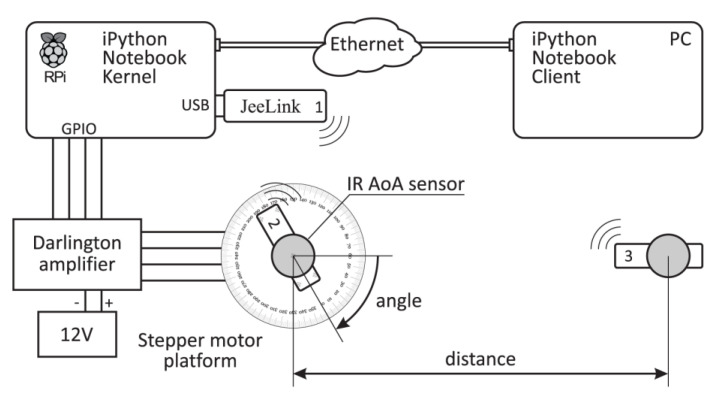
AoA sensor calibration system [36].

The solution to the estimation error induced by the unique radiant sensitivity of each phototransistor was to implement an automated calibration platform (Figure 3) and to use it to record true radiant sensitivities for all transistors like the one shown in Figure 2. The central controlling part of the platform is the calibration server implemented as a RaspberryPi computer running the iPython Notebook kernel. Controlling software communicates with sensor nodes via a connected JeeLink sensor node and controls the rotation of the stepper motor via general-purpose input/output (GPIO) connectors and the power amplifier. The test platform is fully manageable from a personal computer using the iPython Notebook client, i.e., any Internet browser.

The sensor network part of the platform consists of three nodes:

- The JeeLink node, in the scheme labeled as node 1, serves as a gateway: to send commands to nodes 2 and 3, and to receive measured data from node 2.
- Node 2 is mounted on a rotating platform and attached to the sensor being calibrated.
- Node 3 is an infrared transmitter, i.e., it serves as a controlled IR radiation source with known distance and AoA relative to node 2.

For each step of the stepper motor, node 1, attached to the calibration server, triggers a flash of the IR diodes on the node 3 and, at the same time, measurement is taken by each of the 12 IR phototransistors on the sensor attached to the node 2. Measured data is then transmitted back to node 1 and stored on the client computer.

After the measurement has been made, the stepper motor rotates one half-step, or in this case for 0.9° in the clockwise direction, and the procedure repeats for all 360°, i.e., for 400 half-steps. The obtained data consists of a true AoA taken from the known position of the stepper motor and 12 × 400 IR measurements taken from 12 phototransistors for each of 400 different AoA.

Unlike the typical IR transmission (e.g., with remote control device), wherein the signal is modulated in order to separate it from the ambient light, in our case only a DC signal is used. We did not consider modulating the emitted signal because, in our solution, AoA estimation relies exclusively on the relative strength of the signal received on the sensor’s phototransistors.

Possible MP effects were not taken into consideration during the calibration procedure. Namely, we did not experience any unexpected issues in this matter, as long as the sensor or the transmitter was not too close (few centimeters) to some reflective object. In all other cases, the LOS component showed to be a predominant part of the received signal, and, as such, it is de-facto exclusively used for AoA estimation. Our calibration platform was placed in the center of the room, away from the walls and other obstacles, so we can fairly assume that there was no significant MP effect on the sensor calibration.

#### 2.1.3. Estimation Algorithm

To be able to estimate the angle of arrival, the first observation that needs to be made is that the phototransistor readout presents the sum of the IR irradiance from the transmitting node and the ambient. To filter out ambient radiation, the sensor needs to make a measurement at the moment in which all IR transmitters in the range are off. In the following text, all phototransistor readouts are considered to be already filtered, i.e., subtracted by measured ambient radiation.

**Figure 4:**
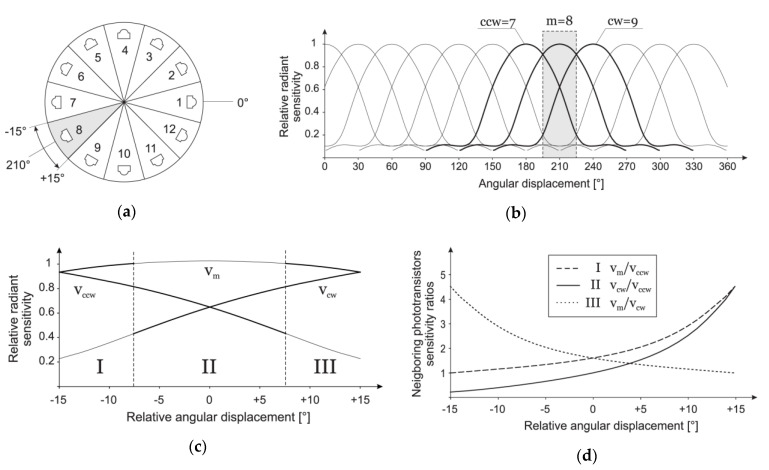
(**a**) AoA sensor layout; (**b**–**d**) diagrams used in the estimation algorithm, obtained from nominal phototransistor sensitivity as defined in the datasheet.

The second observation can be made from real (Figure 2) and nominal (Figure 4b) phototransistors array radiant sensitivity characteristics: for any given angle, only three phototransistors have their output above the level that can be used for the AoA estimation. Therefore, the estimation algorithm selects three phototransistors with the highest output. Since those three phototransistors are always successive and the middle one has maximal output with the following two being its counterclockwise and clockwise neighbors, their outputs are marked as *v_m_*, *v_ccw_*, and *v_cw_*, respectively.

The third observation is that absolute values of the signal irradiance readout cannot be used as is, since they are dependent on the unknown distance between sensor and transmitter. The upside is the fact that the ratio of their values does not depend on distance; thus, the following three values can be used: *v_m_*/*v_ccw_*, *v_m_*/*v_cw_*, and *v_cw_*/*v_ccw_*.

Once phototransistors indices *m*, *cw*, and *ccw* are selected, further AoA estimation is performed in the range [−15°, 15°], relative to the orientation of phototransistor with the maximal value *v_m_*. After measurement data are obtained, the estimation is performed by selecting one ratio and matching its value with calibration data. The selection of the ratio that is used in the estimation depends on the segment of the given range in which AoA is being estimated, and this itself can be estimated using the default *v_cw_*/*v_ccw_* ratio.

The criteria for the selection of the ratio (from three possible ratios *v_m_*/*v_cw_*, *v_m_*/*v_ccw_*, and *v_cw_*/*v_ccw_*) is twofold: (1) expected absolute values that are used in the ratio need to be as high as possible (Figure 4c), and (2) the rate of change, i.e., the absolute derivative of ratios, should be as high as possible, as well (Figure 4d). Using these criteria, the estimation range is divided into three segments, marked as I, II, and III and ratios used are *v_m_*/*v_ccw_*, *v_cw_*/*v_ccw_*, and *v_m_*/*v_cw_*, respectively.

Finally, calibration measurement data is used to calculate all three ratios for any given angle. Strictly speaking, for each of 12 different sections (Figure 4a), i.e., for each triplet of neighboring sensors (*m*, *ccw*, *cw*), calculated ratios from measured values are fitted with polynomials and only coefficients of corresponding polynomials are stored for the estimation. Since all polynomials are monotone (Figure 4d) in the segment they are used, the estimation algorithm itself is reduced to a simple binary search.

**Figure 5:**
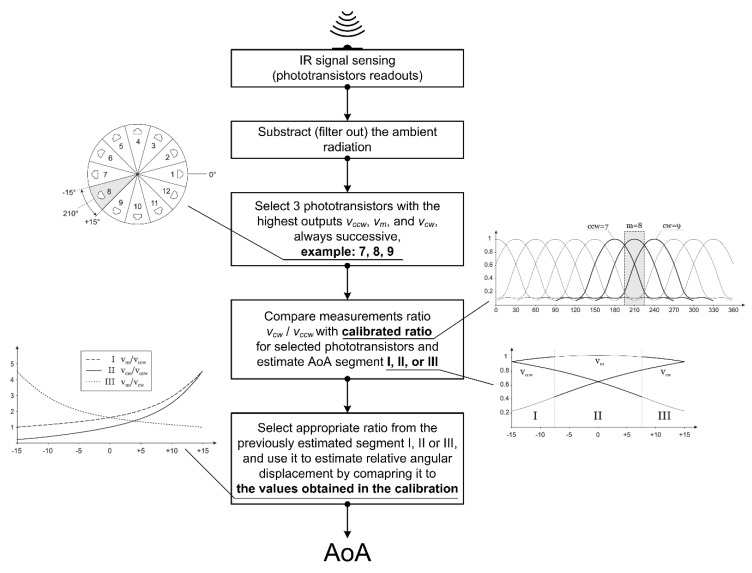
The flowchart of the proposed AoA estimation algorithm.

In order to systematically visualize the required steps in the proposed AoA estimation algorithm, we additionally present the corresponding flowchart (Figure 5).

**Figure 6:**
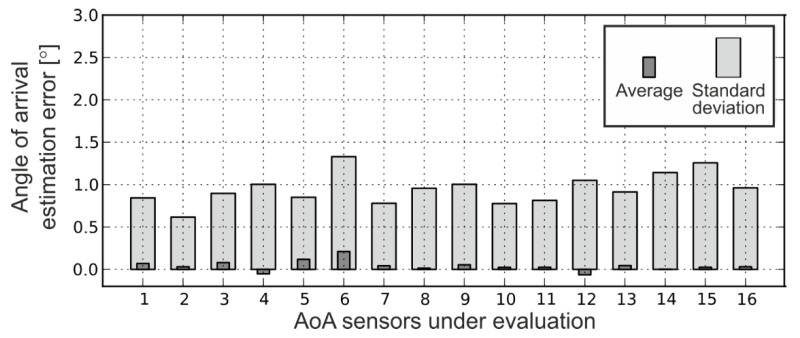
Angle of arrival estimation error for 16 different sensors.

Custom calibration and the described algorithm were used in the evaluation of 16 different AoA sensors. As shown in Figure 6, this approach reduced the standard deviation of the estimation error to around 1°. The main drawback of the IR AoA sensor is its reduced range: We have been able to capture IR signals and estimate the AoA with a distance between transmitter and sensor up to 4 m. On the other hand, since the algorithm for the AoA estimation utilizes the ratio of the detected IR levels on different phototransistors, the range could be further increased simply by using more powerful IR transmitters and AD converters with a variable reference voltage.

### 2.2. Showcase Application: Supermarket Navigation

Our showcase application assumes enriching in-situ shopping experience by providing cart localization and navigation services within supermarkets aisles. Supporting this context is motivated by current customer demands, as well as by recent trends that suggest increasing involvement of modern technologies within the shopping process, i.e., smart shopping [37]. According to a 2016 study by ECE [38], a German project manager for shopping centers, end users want to utilize digital services in brick-and-mortar stores. The study showed that, among people younger than 40, every third person needs some sort of in-store guidance system. Billa, one of the most famous Austrian supermarket chains, has a mobile application that utilizes Bluetooth Beacon technology for providing location-based promotions in 11 selected shopping venues. French multinational retailer Carrefour, as well as Target, the second-largest discount store retailer in the United States, both invested in VLP-based indoor navigation prototype solutions, to assist their customers in finding what they are looking for more easily [39,40].

A supermarket is a typical example of a dynamic indoor space with dense obstacles. As noted in the Related Work subsection, there is a large number of approaches for solving indoor localization problem in such a context. Most of them are tackling a hard problem with complicated multipath signal propagation. Those approaches are either using different heuristic methods to overcome the lack of closed-form solution for NLOS propagation or trying to ensure simple LOS propagation by dense dissemination of beacons, demanding complicated and high-cost installation.

**Figure 7:**
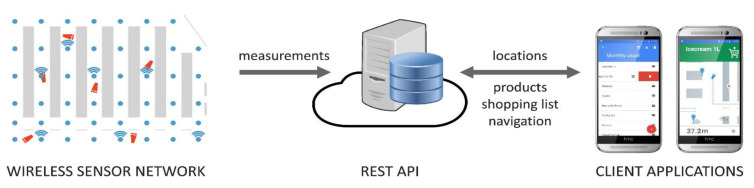
Navindo indoor navigation system. (1) Wireless sensor network with nodes deployed at fixed locations (i.e., above aisles in the supermarket) and simple IR transmitters (tags) on mobile objects that are being located—carts. (2) Application programming interface (API) that provides support for managing both the location data and the information about the target navigation area. (3) Client mobile applications used for accessing, managing, and visualizing location data.

The key aspect of the proposed system, code-named Navindo, is to integrate readily available and inexpensive technology, such as the AoA IR sensor described in the previous section, with state-of-the-art low-power communication technology provided by modern wireless sensor nodes. In general, Navindo demonstrates how to overcome drawbacks of any system in need of LOS propagation using inexpensive, autonomous, and easily deployed wirelessly connected nodes. The benefit is twofold: (1) the system is accurate since there is an abundance of the high-quality LOS signal throughout the environment, and (2) the system is simple and inexpensive to install and maintain. Navindo consists of 3 components, as shown in Figure 7.

#### 2.2.1. Wireless Sensor Network

The key piece of the proposed solution is a wireless sensor network that extends the usable region for high-precision LOS mobile node localization based on the IR AoA sensor. The main purpose of the wireless sensor network is to provide infrastructural support for real-time navigation in the supermarket context. Having in mind the typical organization of the brick-and-mortar shopping venue, consisting of shelves and corridors, a straightforward WSN topology is assumed which relies on placing sensors above the carts’ movement area. Hence, nodes equipped with IR AoA sensor are positioned directly above aisles, every few meters, preferably clipped onto light sources that are usually hanging low enough to evenly illuminate products on the shelves. Each node with its IR AoA sensor can track the IR signal along the aisle with the standard deviation of error below 1°, as described in the previous section.

Additionally, each cart is equipped with a simple IR transmitter and, currently, its sole function is to transmit its ID number allowing both identification and localization from the sensor nodes side. The benefits of leaving RF transceiver out of mobile nodes are twofold: (1) cart is kept as simple as possible, and (2) the number of RF transmitting nodes is reduced, consequently reducing packet collisions and decreasing latency and throughput of the WSN.

Deployment of the previously described system is simple and straightforward; thus, the cost of the related installation is expected to be low. Since the density of the WSN directly determines the price of the system installation, the overall budget can be predicted based on the required number of WSN nodes and the expected number of the IR transmitters (carts). In the following text, we present empirical results that suggest that one sensor node per every 3 m of the corridor represents a suitable density. Furthermore, we introduce the possibility to reduce system operating costs even more, by proposing energy harvesting scenarios.

**Testbed.** In our testbed, we used commercially available JeeNode nodes based on Atmel ATmega328p microcontroller and RFM12B radio module. The radio module is using an 868 MHz ISM frequency band, with a data rate of 49.261 kb/s, and an indoor range from 10 to 20 m. Mobile nodes (carts) are equipped with simple IR diodes and the radio module turned off. Sensing nodes, positioned above corridors, are equipped with the proposed IR AoA sensors. A single gateway node was equipped with the EtherCard extension module based on the ENC28J60 chip and connected to the Internet using a wired local area network.

WSN communication protocol is based on the JeeLib library [41] with an address space of 250 groups with 30 regular nodes each. The media access part of the protocol implements a simple CRC-16 algorithm for error detection but with no collision avoidance. Thus, in the proof-of-concept laboratory setup, consisting of no more than 10 nodes, we used a simple centralized algorithm based on iterative queries. In this algorithm, the gateway node was also a master node, i.e., the one issuing all queries, waiting for response messages, and forwarding the corresponding data. This node was sequentially querying other nodes for fresh AoA measurements: The response message payload was a pair of values consisting of the ID of the transmitter and the AoA measurement. After each query, the gateway node simply forwarded the measurements to the localization server API.

In this setup, sensor nodes are continuously listening for IR signals and storing all measurements until they receive a query from the master node. This way all RF collisions were avoided with an obvious issue of poor scaling of the network, but that was an inherent drawback of the selected platform.

Our future work plan includes upgrading the platform to the custom Texas Instruments CC2538 or CC2650 SoC based node. The plan is to implement an algorithm on top of the Contiki operating system and use its protocol stack consisting of state-of-the-art protocols, such as IEEE 802.4.15, 6LoWPan, RPL (Routing Protocol for Low-Power and Lossy Networks), and CoAP (Constrained Application Protocol), thus enabling large address space that can host a much larger network, multi-hop routing, low power operation, etc.

**Figure 8:**
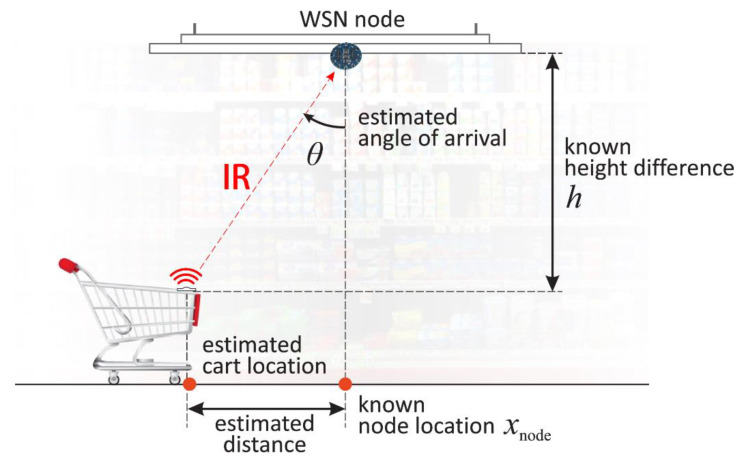
Cart location can be estimated in 1D (along the aisle) using AoA measurement in combination with the prior knowledge of the wireless sensor network (WSN) node location and height difference between node and IR transmitter. In this setup, the AoA sensor is rotated in order to estimate the angle of arrival in the x–z plane. The location of the cart is calculated using an estimated angle, a priori known AoA sensor position, and simple trigonometry relation: $x_{cart}=x_{node}+h\cdot \tan\left(\theta \right)$.

**Measurement protocol.** Every measurement is initiated by the IR transmission from the node placed on the mobile cart, after which sensing WSN nodes in its range detect IR signal and estimate AoA, thus enabling estimation of the cart location as shown in Figure 8. To unambiguously identify the transmitting cart, every IR transmission is prefixed with the cart ID encoded with a slightly customized NEC IR protocol. This protocol is the de-facto standard and, as such, used by many consumer electronics, mostly for remote control. In our setup, the ID is an 8-bit number allowing identification of 256 different carts. The duration of the IR transmission is the sum of the modulated ID prefix and continuous IR signal used for AoA measurement. Using the NEC protocol for encoding ID prefix takes 40.5 ms, and the measurement signal takes an additional 25 ms, resulting in a total transmission duration of 65.5 ms. After the detection of the IR signal, the sensing node decodes the mobile node ID and then, from a measurement of the incoming IR signal on 12 IR transistors, estimates the angle of arrival.

IR AoA sensor, in its current form, can detect and measure AoA of just one IR transmitter at a time. The number of transmitters in the low sensor range is relatively small and transmission duration takes only 65.5 ms, with the potential to be further reduced. Therefore, to handle multiple transmitters, we opted to use Time Division Multiplexing (TDM). Since we wanted to keep transmitters simple and offline, i.e., with no RF communication, we did not introduce additional synchronization overhead required for TDM. Consequently, the collision of IR packets from nearby carts presented a potential issue. Each cart is usually in the range of only 2 to 3 different sensor nodes, so we chose to solve the collision problem using simple transmission delay randomization. In our prototype system, IR signals from the carts were transmitted with a randomly chosen delay between transmits, ranging from 0.5 s to 1.5 s. Collision probability depends on the number of carts in the range of the sensor.

**Figure 9:**
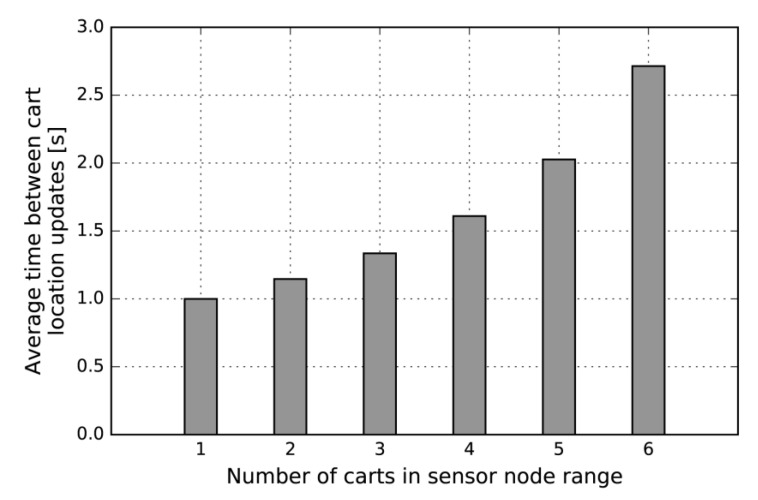
The average time between cart location updates depending on IR package collision probability or, more precisely, on the number of carts in the sensor node range. With a standard deployment density of 1 sensor every 3 m, there is a high probability of multiple sensors in the IR range of the mobile node, further reducing latency.

An additional advantage of using IR signals is directed radiation (towards sensors installed above corridors) and relatively small signal range. This property significantly reduces the collision probability, thus avoiding the need for time synchronization of the transmitters, given the small duty cycle required for transmitting. Thus, in our analysis, we considered 1 to 6 moving carts in the range of one AoA sensor. The resulting average times between cart location updates are presented in Figure 9.

We did not consider FDMA (Frequency-Division Multiple Access) or CDMA (Code-Division Multiple Access) instead of TDM, simply because we think that the average time between cart location updates, as provided with TDM, is appropriate for the target context. Specifically, we find the “in-aisle crowding scenario”, wherein more than 4 shopping carts are visible to only a single AoA sensor, less likely to happen. Hence, for a typical WSN topology (1 sensor per every 2–3 m) and a typical shopping scenario, we expect cart location updates to take place every 1–1.5 s. We find this frequency to be suitable for the target localization service, i.e., for real-time navigation support within supermarket corridors.

**Localization algorithm.** To be able to estimate the location of the mobile node, using just AoA measurement, the position (location and orientation) of sensing nodes must be known at installation time. Thus, the localization algorithm can map each node *i* to the following tuple (*x_i_*, *y_i_*, *h*, *φ_i_*), that is, the location of the node in 3D: *x_i_*, *y_i_*, height *h* (usually the same for all sensors), and the orientation of the sensor in *xy* plane, *φ_i_*. This is a reasonable demand which introduces additional benefits: simplifying topology control, designating node roles, and setting transmission parameters, such as timings and signal strengths.

**Figure 10:**
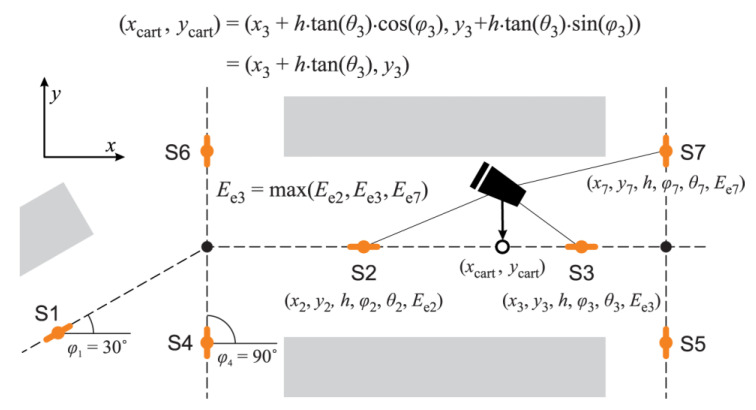
Sensing nodes S1–S7 are placed on the edges of the aisles graph (dashed line). After the IR transmission from the cart took place, nodes S2, S3, and S7 performed measurement and AoA estimation. Measured irradiance on the node S3 was the highest so the cart was localized using estimated AoA *θ*_3_ and the position of the sensing node S3 in the Equation (1). The orientation of the node S3 is *φ*_3_ = 0°; thus, the estimated location is ($x_{3}+h\cdot \tan\left(\theta_{3}\right),\;y_{3}$).

Another prerequisite for the localization, and later for the navigation, is the aisles graph, seeing that all estimated locations are subsequently mapped onto its edges. The aisles graph represents a navigation layer of the supermarket map. Generally, WSN nodes are positioned on the edges of the aisles graph and are oriented in the direction of the edge they reside on, as can be seen in Figure 10.

As described previously, after each IR transmission the AoA measurement and estimation are initiated on every WSN node in the range. If the measurement was successful, cart ID, estimated AoA, and the maximum measured irradiance level are all sent to the gateway node as soon as possible. On the reception of measurements, the gateway simply forwards them to the localization server using REST API. The localization algorithm itself is executed on the server. Input for this algorithm is the set of measurements initiated by the same IR transmission. Measurement of the sensing node *i* is defined by the tuple (*i*, *θ_i_*, *E_ei_*), that is, ID of the sensing node, estimated AoA *θ_i_*, and maximum measured irradiance *E_ei_*.

The localization algorithm is consisting of the following three steps:

1. Select measurement → from all measurements in the set, pick the one with the highest maximum measured irradiance *E_ei_*. This step is based on the simple heuristic assuming that the highest irradiance measurement correlates with the lowest distance between the transmitter and the sensor, and, more importantly, with the lowest geometric dilution of precision (GDOP).
2. Estimate location from selected measurement → selected measurement, along with the position of the corresponding sensing node, is used in the simple equation to estimate cart location:
  (1) $$\left(x_{cart},\;y_{cart})=(x_{i}+d\cdot \cos(\varphi_{i}),\;y_{i}+d\cdot \sin(\varphi_{i})\right)$$
  where *d* is the Euclidean distance of the estimated projection of the cart position onto the line passing through the sensing node in the direction of its orientation *φ_i_*:
  (2) $$d=h\cdot \tan(\theta_{i})$$
3. Estimate the location on the aisles graph → find the nearest point on the aisles graph edge from the estimated location. This step is usually straightforward since the aisles graph itself is constructed according to the positions of the sensors; thus, the distance of the estimated location from the graph tends to be zero. As will be described later, this mapping of the location to the aisles graph edges is important for the shortest path navigation to the products on the shelves.

**Figure 11:**
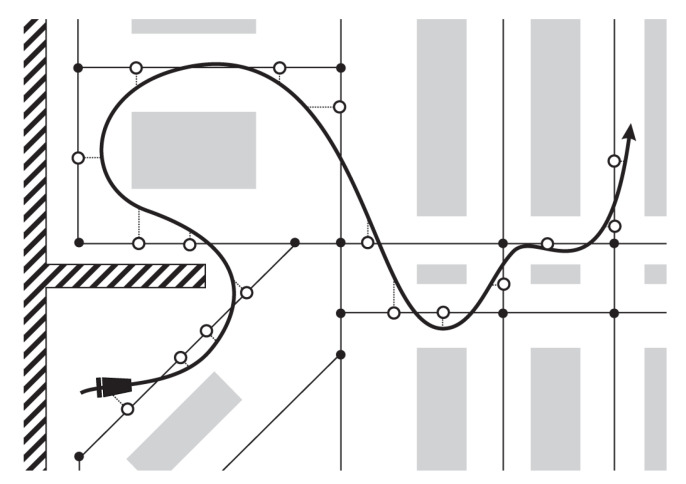
Localization strategy based on aisles graph. Vertices of the aisles graph are marked with • (black dot), and estimated locations are marked with ◦ (white dot). WSN nodes are not visible. All estimated locations reside on the aisles graph edges. The cart is localized after each IR transmission.

Using the described algorithm, the real trajectory of the mobile node is estimated as the sequence of locations placed on the edges of the aisles graph. Each estimated location is the result of the successfully received IR transmission as presented in Figure 11.

As stated previously, in the presented localization solution, the position (both the location and the orientation) of sensing nodes must be known at installation time. Consequently, while setting the WSN topology at the target indoor environment, sensors should be installed precisely, following the specified distance and in the direction of the aisle-graph edges. If a particular sensor is not oriented correctly (with a certain angular deviation from the aisle-graph edge), then the associated error will propagate in absolute amount and thus affect the location estimation correspondingly.

The localization accuracy of the WSN described above, which utilizes novel IR AoA introduced in this paper, is thoroughly tackled in the Results section. However, for the sake of completeness, we continue this part of the paper by presenting the remaining parts of the Navindo indoor navigation system.

#### 2.2.2. Server and API

The part that connects all Navindo components in one system is the server and its front-facing application programming interface (API). It is utilized both as permanent storage of measurement data gathered from the wireless sensor network and for the implementation of the business logic for all client applications. The chosen software stack includes Debian Linux OS, PostgreSQL database, Nginx web server, and Gunicorn application server. API was implemented using Python programming language, Django, and Django Rest Framework package. It implements several functions, such as:

- WSN measurements retrieval and storage,
- WSN node layout management,
- Cart location estimation and update,
- Product locations management,
- Store layout management,
- User signup and login,
- Token-based authentication,
- User cart registration,
- Shopping lists management,
- Shopping list based shortest path navigation (directions).

**Figure 12:**
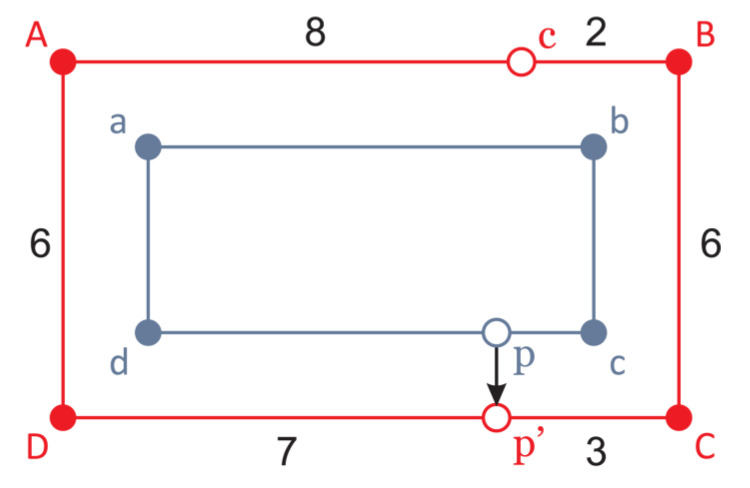
Shelves and aisles graphs: {*a*, *b*, *c*, *d*} is the set of vertices of the shelves graph with the product location *p* on the edge (*c*, *d*) and {*A*, *B*, *C*, *D*} is the set of vertices of the aisles graph with cart location *c* on the edge (*A*, *B*). The product location is mapped to the aisles graph as *p’* and the shortest path from cart to product is (*c*, *B*, *C*, *p’*) with length 11.

On the server, each supermarket is modeled using two graphs: the aisles graph and the shelves graph (Figure 12). Carts and WSN nodes are located on the edges of the aisles graph, and products are located on the edges of the shelves graph. Each product can be mapped from the shelves graph to the nearest point(s) on the aisles graph. This way all navigation algorithms, such as the shortest path and the traveling salesman, are performed on the aisles graph, while locations of all objects in the 2D coordinate system are preserved. For the simulation purposes, an existing retailer’s webshop with more than 20 thousand items was automatically scraped, and obtained items were algorithmically distributed on the shelves graph edges.

#### 2.2.3. Client Applications

**Figure 13:**
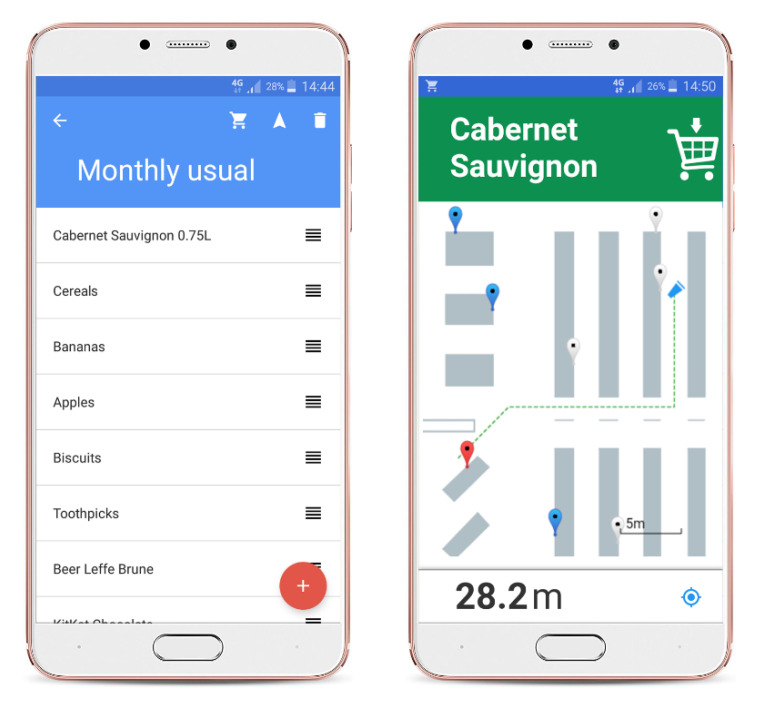
Mobile application screenshots. Shopping list editor screen on the left and the navigation screen on the right. The dotted line on the navigation screen represents the shortest path on the aisles graph from the current estimated cart location to the location of the next product in the shopping list. The more detailed view of the corresponding aisles graph is presented in Figure 11.

In our proof-of-concept solution, we developed a client mobile application for Android devices that encompasses shopping list utilities and indoor navigation services (Figure 13). After the initial registration with the Navindo system, the user is allowed to manage shopping lists by making use of the product database.

Once the user enters a supermarket and gets a shopping cart, navigation services can be enabled. In the mobile application, this is done by cart registration activity wherein the user is required to enter the cart ID (information provided on the cart itself). Navindo system provides the user with two basic options for routing within a store: (1) using shortest paths to all products from the list in the predefined order and (2) reordering the shopping list automatically to generate the global shortest route for picking all products (an instance of Traveling Salesman solution).

Navigation activity is designed in a way to resemble Google Maps user experience (Figure 13). As the user is moving inside the store, the position of the shopping cart is updating in real-time. If a smartphone running the Navindo application is equipped with the compass sensor, the current orientation of the shopping cart will be visualized on the map, as well. To utilize this option in full scale, the smartphone device should be mounted on the trolley, for which simple holders can be used (similar to car phone mounts).

Once all products from the shopping lists are collected, the mobile application routes the user to the counters zone, thus completing the navigation assist. Localization context can therefore be deactivated by unregistering the related cart ID.

**Figure 14:**
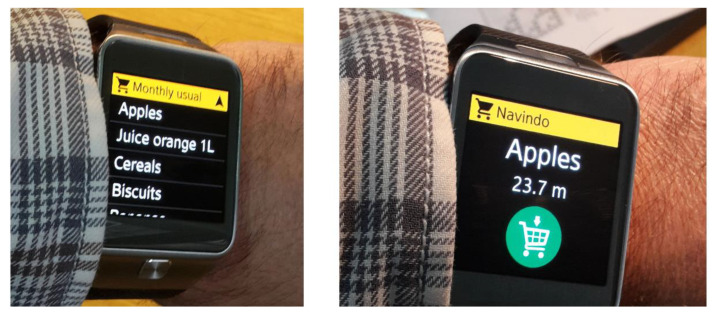
Smartwatch application.

To further augment location-aware shopping, as well as to demonstrate extra benefits of ubiquitous computing in the supermarket settings, we also developed an accompanying Navindo smartwatch application. According to the inherent limitations of the smartwatch I/O capabilities, only a subset of smartphone application functions is provided. The related use cases are shown in Figure 14. Samsung Gear 2 watch, running Tizen OS, was used in our proof-of-concept solution.

#### 2.2.4. Prospective IoT Services

Along with the on-site customers, using mobile applications on their gadgets (smartphones and smartwatches), retail management represents another user group that can substantially benefit from the Navindo system. Since the information of both the products and the gathered locations are available in real-time, extensive data analytics can be performed and subsequently used in order to boost efficiency and sales numbers. According to the collected location-based information, a detailed insight can be provided for the following cases: customers movement routes within a store, time of dwelling in the particular zones (heat maps), crowding scenarios, products purchase frequencies, customer feedback to promotional offers, a correlation between product locations and purchase volume, etc. Such analysis could further lead to strategic decisions about promotional offers, personalized marketing, shopping gamification (coupons and prizes), product placement and exposure, store layout optimization [42], human resources planning, and supermarket activities in general.

## 3. Results

In this section, we present the testing procedures and the results concerning the localization performance of the proposed AoA-sensor-based WSN solution. We investigated the localization accuracy of the WSN by performing four different evaluations as follows:

- **E1:** Static-1D (empirical, laboratory settings),
- **E2:** Mobile-1D (empirical, laboratory settings),
- **E3:** Static/Mobile-2D (empirical, laboratory settings),
- **E4:** Large-scale Mobile-2D (simulation).

**Figure 15:**
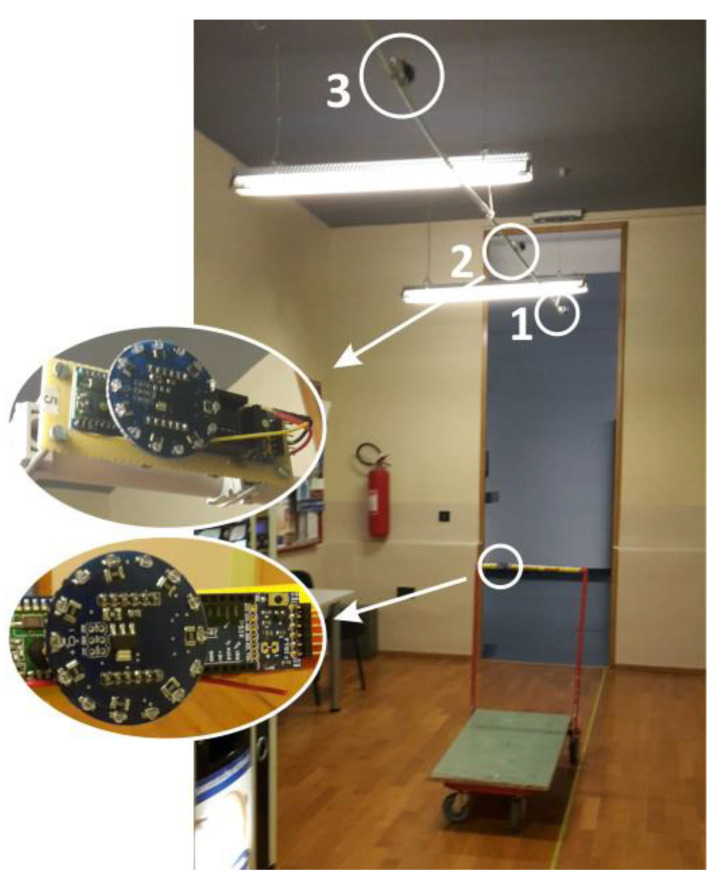
E1-Testbed: numbered nodes (1, 2, 3) are equipped with an IR AoA sensor, and on the handle of the cart is an IR transmitter. Its design is similar to the sensing node since the IR AoA sensor has IR diodes on the opposite side of the IR phototransistors.

**Figure 16:**
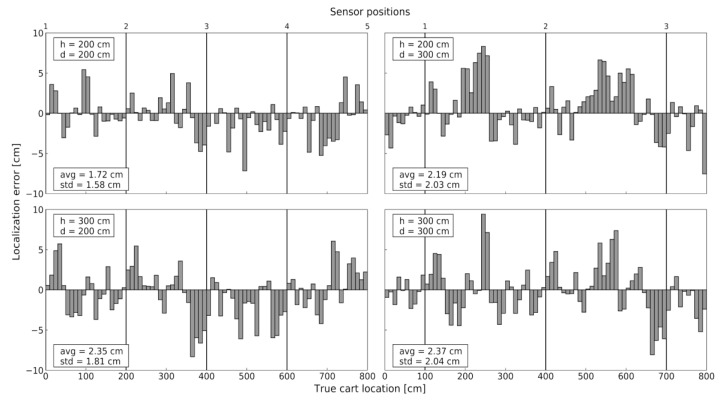
Localization error experimentally measured in the 8-m-long corridor for every 10 cm. Distribution of nodes with IR AoA sensor *d* was one in every 2 and every 3 m, left and right column, respectively. The height of the sensing nodes above the IR transmitter *h* was set to 2 and 3 m, top and bottom row, respectively. As can be seen from the presented results, in all scenarios, localization error did not exceed 10 cm.

In the first experiment (E1), we examined localization accuracy considering, altogether, four possible sensor deployment patterns. The effect of the WSN density was assessed using a proof-of-concept setup with sensing nodes positioned every 2 or 3 m, along the corridor (Figure 15). The height of the sensing nodes above the IR transmitter level was set to 2 m and 3 m. We opted for a 2 × 2 experimental design, pragmatically considering two discrete values for both sensor spacing and sensor height, having in mind the current signal range of the proposed IR AoA sensor. For every combination of sensor spacing *d* and height *h*, the cart was moved down the corridor and localized every 10 cm. The reference trajectory was a straight 8 m line along the aisle, and the cart was kept static in each measurement point (thus the Static-1D label). Resulting localization errors are presented in Figure 16, revealing the centimeter-level accuracy of the proposed solution for the corresponding setup.

According to the obtained results, we can reasonably recommend WSN density with 2–3 m sensor spacing, as well as sensor placement onto the lighting infrastructure. It is assumed that supermarket lights are placed above the aisles, 2–3 m above the IR transmitter level, which is typically a case in order to evenly illuminate all shelves. In the described experiment E1 all measurements were performed with the static IR transmitter. However, in the real-world scenario, the transmitter is mobile and, due to the described transmission delay, the real-time localization error depends on movement speed. To evaluate the localization error of the mobile transmitter we performed the second experiment (E2).

**Figure 17:**
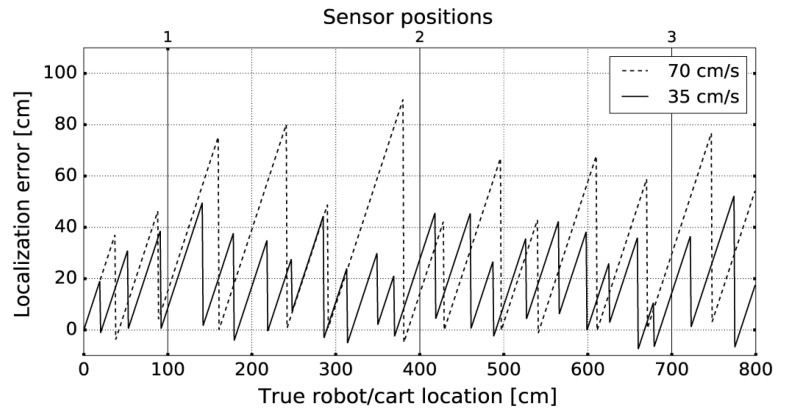
Localization error of the mobile transmitter for two different velocities experimentally measured in the 8-m-long corridor. The height of the sensing nodes was set to 3 m above the transmitter. The transmission delay is uniformly distributed between 0.5 s and 1.5 s. It can be seen that error is significantly and rapidly decreasing after the IR transmission events. Although varying, localization error showed to be bounded below 50 cm for 35 cm/s and below 90 cm for 70 cm/s. The localization error in this context represents a displacement of the estimated location from the real location in 1D (i.e., the displacement on a robot trajectory line); thus, it can be negative.

In the experiment E2, we used an IR transmitter attached to the Pioneer AT-3 mobile robot platform. The robot was set on a straight 8-m-long trajectory, representing the cart movement along the aisle, with constant velocities of 70 cm/s (maximum robot speed) and 35 cm/s. Three AoA sensors were distributed along the trajectory every 3 m and were positioned 3 m above the IR transmitter on the robot. Obtained localization errors are presented in Figure 17.

As expected, the error is dependent on movement speed since the time delay between two consecutive location updates is kept in the constant range. Location update is performed immediately after the IR transmission event, reducing the localization error to the level obtained in the static context. We find these results to be suitable for supermarket navigation, considering the usual movement speed of customers and the context in which fine-grained localization is needed only when the customer is moving slowly.

**Figure 18:**
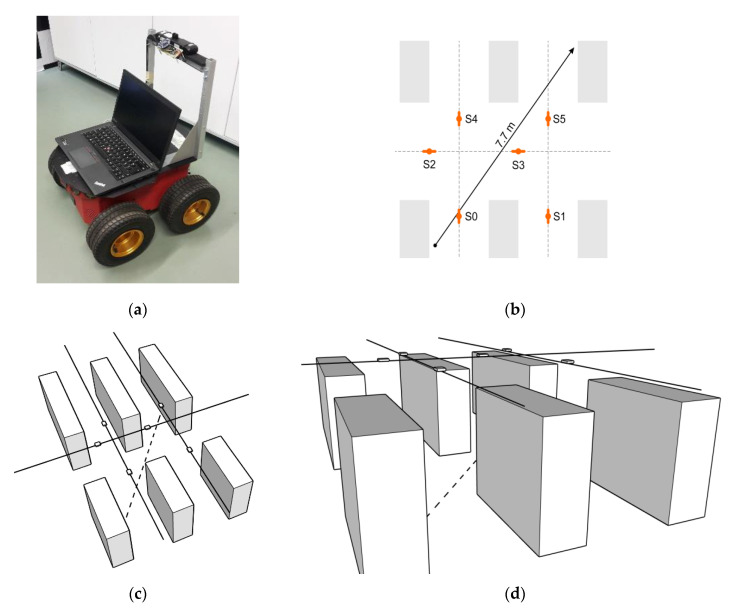
(**a**) Pioneer AT-3 mobile robot platform with IR transmitter attached to its handle; (**b**) Static/Mobile-2D experiment setup (WSN topology and movement trajectory used in E3); (**c**,**d**) 3D models of the E3 setup: six AoA sensors are placed above the shelf mock-ups made of cardboard boxes.

Having in mind that the cart’s true position can be outside of the aisles graph, the goal of the third experiment (E3) was to evaluate the localization accuracy of the proposed solution in a more realistic Mobile-2D context. This time we set the robot (Figure 18a) on a 7.7-m-long path within the specially designed topology wherein 6 sensors were set in a 2D mesh (Figure 18b). This topology determines the corresponding aisles graph on which the robot can be localized by our solution. All sensors were positioned 3 m above the IR transmitter level. In order to make the experiment setup similar to the supermarket scenario, the given route was positioned between the shelf mock-ups made of cardboard boxes. Altogether, six “shelves”, each 220 cm high, were thus used to simulate the target context (Figure 18c,d). Although there were three people in the laboratory during the experiment (two experiment administrators and a robot operator), we did not formalize any intentional user movement in that space. Hence, the MP effects were not specifically analyzed.

**Figure 19:**
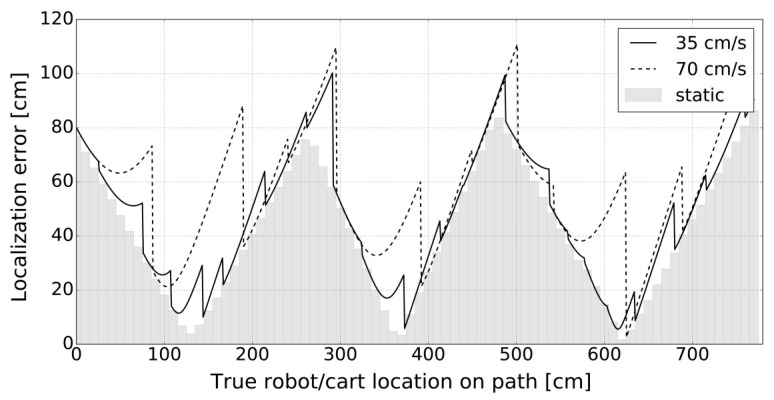
Localization error obtained in E3. Considering the low Static-1D error from the E1 experiment, we can conclude that the Static-2D localization error mainly originates from the distance between the cart true location and the aisles graph and, to a lesser extent, from the AoA measurement error. The Mobile-2D localization error additionally includes a component related to the movement speed and the IR transmit time delays as examined in the experiment E2.

Given that the used AT-3 robot can easily maintain a constant speed on the straight line, we opted for the linear trajectory once again, which allowed simple calculation of fine-grained ground truth 2D locations in real-time. The robot traveled along the same trajectory with constant velocities set as in the Experiment E2 (70 cm/s, 35 cm/s). Additional static localization, like in the E1, was performed with a 10 cm resolution on the same path. Localization errors obtained in the given E3 setup are presented in Figure 19.

The localization error in E3, as opposed to E1 and E2, contains an additional component (distance from the aisles graph; thus, the localization accuracy in the 2D mobile context is expectedly lower.

**Figure 20:**
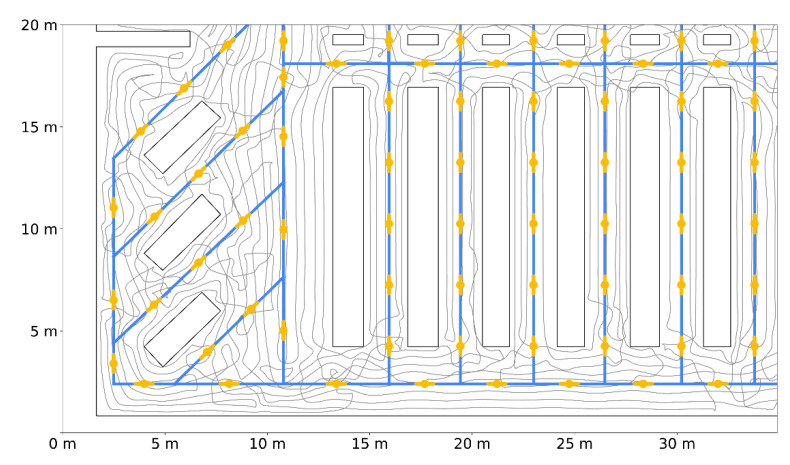
Large-scale Mobile-2D simulation: a part of the supermarket topology with IR AoA sensors, aisles graph, and cart trajectory.

Finally, to assess the general localization error of the proposed solution, we carried out a simulation (E4) based on a large-scale movement trajectory (Figure 20).

For an 80 × 38 m supermarket layout, we designed a WSN topology with exactly 301 IR AoA sensors that were simulated according to the real characteristics of the 16 prototype sensors. We distributed simulated sensors along the virtual corridors so that every location in the supermarket has at least one sensor in the IR transmitter range. The corresponding aisles graph was constructed according to the store layout and the designed WSN topology. In the described setup, we inserted a large-scale cart trajectory that uniformly covers available movement space for the given layout. This 5000-m-long trajectory was obtained using the ergodicity-based coverage algorithm [43], targeting the homogeneous area coverage of the simulated cart locations. We simulated shopping cart movement along the given trajectory using three speeds: 35 cm/s, 70 cm/s, and 140 cm/s. Ground truth locations from simulated trajectory were compared to estimated locations provided by WSN, and localization error was thus inspected. Table 3 summarizes the results of the E4 evaluation.

In all empirical evaluations (E1, E2, and E3), we measured the ground truth information manually. Sophisticated equipment was not at our disposal, and all the measurements were obtained using a simple laser pointer and a laser distance measuring device. The same gadgets were used for setting the experiment scenarios (sensor placement in E1, E2, and E3, as well as shelf mock-ups layout in E3). For static localization purposes, ground truth measurements were taken at the exact positions for which the system provided the corresponding estimations. On the other hand, for the mobile context, we manually measured only the starting and ending point of the robot movement trajectory, while the other ground truth locations were calculated (according to the constant speed of the robot). Although we did not formally determined the accuracy and precision of the ground truth data, we can assume, given the magnitude of the localization error, that these factors do not significantly affect it. Hence, we consider the effects of ground truth precision and accuracy small enough to be neglected concerning the obtained localization error.

We find obtained localization performance suitable for the supermarket navigation context. Namely, in a scenario where a customer is looking for a specific product on the shelf, and/or wants to get visual feedback about the cart location within a dense supermarket map, localization on the aisles graph will provide adequate information. Put differently, in a typical supermarket layout with 2–3 m wide corridors, localization on the aisle centerline, along with the cart orientation visualization, should be sufficient for user-friendly blue-dot navigation. Localization error of the proposed solution generally depends on the indoor venue layout (e.g., wider corridors), as well as on the movement trajectory, but appropriate WSN topology can be utilized to mitigate this error.

Due to the simple design and favorable position of nodes, there is an additional opportunity of using energy harvesting as the main power source, thus limiting or even avoiding network energy maintenance costs. Stationary nodes could be positioned onto the lights which would open up the possibility of using photovoltaic cells to power them. Simple IR transmitters placed on carts only need to transmit when moving, so they could be powered using energy from the cart motion (i.e., wheels rotational energy).

## 4. Discussion

As shown within the simulation (experiment E4), which considers the effect of the proposed localization method in a larger space (supermarket level) with a topology that includes corridors and high shelves, for the obtained localization error level (0.6–1 m, depending on the cart speed) it is sufficient to provide 301 AoA sensors on a gross area of 3040 m^2^. We consider such a WSN density (~1 sensor per 10 m^2^) to be a suitable solution, given the trade-off between the cost estimation of the related infrastructure and the provided localization accuracy in the target context. Specifically, knowing the production costs of both the proposed IR AoA sensor (~USD 13) and the proposed IR transmitter (~USD 9), we can assume the total cost for an 80 × 38 m retail venue with exactly 100 shopping carts: USD 4.813. We find this amount to be a rational investment for supermarket management, seeing that the shopping experience could be considerably enhanced via localization and navigation services. A more densely deployed sensors would allow even better localization accuracy (as shown in experiment E1), however, according to the already mentioned cost-accuracy trade-off, the idea is to use a reasonable number of sensors within a given indoor environment. As stated at the end of the previous section, the infrastructure maintenance costs could be furthermore reduced by utilizing energy harvesting (on both the sensor and the transmitter side), which is part of our future work plans.

Regarding the deployment aspects, since the proposed solution localizes the user on the aisle graph (i.e., in the corridors between the shelves with products), the WSN topology design is rather straightforward. Namely, the proposed sensors should be placed above all the aisles in which users are expected to be walking through (and not above the shelves), with a distance of 2 m to 3 m along the corridors (as demonstrated in conducted experiments). Since no cabling is needed for powering and connecting the WSN infrastructure, we assume supermarket lights and cart handles to be a pragmatic choice for placing the IR AoA sensors and IR transmitters, respectively. In most cases, the lighting infrastructure in a supermarket corresponds to the expected WSN topology, as lights are usually placed above the aisles, in a way to evenly illuminate all shelves.

Seeing that the proposed solution, based on the novel IR AoA sensor, enables cart localization with the corresponding error between 0.6 m and 1 m (in the mobile 2D scenario), the question of comparability with other mature solutions with similar accuracy (e.g., RFID-based and BLE-based) arises. When it comes to the RFID-based localization in the supermarket scenario, we are usually considering carts equipped with RFID tags, and RFID readers deployed at the venue according to the given density. As demonstrated in Reference [15], the problematic aspect of the related RFID-based method can be a long measurement time, and, consequently, the need for the cart to remain stationary (up to 2 s) in order to estimate its current location. Furthermore, if a passive RFID system is selected as the backbone for the localization solution, then one must consider the high cost of multiple RFID readers. For example, high-performance RFID readers, such as the *Impinj Speedway RAIN RFID Reader* (used for baggage tracking, retail inventory management, etc.) reaches a price of over USD 1.000 per single unit. On the other hand, BLE beacons seem to be the most competitive hardware in terms of cost estimation, seeing that the average price for a single beacon is around USD 25 (it depends on the manufacturer, the transmission range, and the form factor of the beacon). However, typical localization accuracy within the BLE-based systems is around 2 m, which makes them more suitable for less precise services, such as proximity-based localization and point-of-interest detection.

One can argue that 2 m localization error, achievable via mature RFID or BLE solutions, can represent an adequate accuracy level for cart localization in supermarkets. We agree that such accuracy can be considered satisfactory, but, at the same time, we think that the end-user should, as far as possible, be provided with highly usable localization services, here including seamless and precise navigation support. Hence, one of our goals was to provide a direction-finding interface, similar to that from the well-known navigation applications, such as Google Maps. In this sense, the proposed system supports real-time localization (and, consequently, navigation) wherein the localization error changes dynamically, depending on the cart movement speed and the distance from the aisle graph. We can assume that a larger localization error will not affect (negatively, to a greater extent) the usability of the navigation service when the user searches for the target shelf at a higher speed. However, once the customer reduces the cart speed coming in front of the shelf, where the target product should be found, then a lower localization error can considerably affect (positively) the overall user experience. If the user, while fine-searching for a target product, stops in the middle of the corridor (quite a possible scenario), then the proposed solution can localize the corresponding cart at the decimeter level (as demonstrated in the E1 experiment). In addition, when considering RFID and BLE solutions, we have to take into account the fact that shelves for them are practically “transparent”. This means that 2 m error can imply location estimation in a corridor adjacent to the ground truth, effectively making the respective error fairly larger for the end-user. Conversely, in our LOS system, the shelves represent the obstacles for the IR signal, and, following the aisle-graph model, the cart will be localized in the right corridor.

Regarding the comparison of the proposed IR AoA sensor with the existing IR sensors for localization (some of them are tackled in Section 1.1.2), we think that the results of such a comparison would be difficult to generalize for the target setting. Namely, our localization method depends on the specific spatial context, i.e., the mobile transmitter can be localized on the aisle graph exclusively, which inherently contributes to the localization error. However, we are willing to retain that component of the total localization error, believing that the aisle-graph model represents a suitable trade-off for the supermarket environment.

The showcase application of the proposed indoor localization principle is providing a cart navigation service within a supermarket venue on the aisle level. Mobile applications for smartphone and smartwatch devices are developed, confirming the utility of location-based data provided by the underlying WSN in real-time. The proposed system has a better cost-benefit ratio when compared to competing solutions, considering the tradeoff between required installation and maintenance expenses and achieved localization precision.

To the best of our knowledge, the WiDeo system [9] represents the best competing approach in terms of both the achieved accuracy and expected installation costs. The object being traced using WiDeo does not have to be accompanied by any supplementary device, which is a noteworthy advantage among existing localization solutions. However, according to the reported experiments, WiDeo can trace only five independent concurrent motions without worsening its accuracy which is, according to the authors, “sufficient for a home environment, but not for work environments where a far greater amount of motion is expected”. Moreover, it must be noted that WiDeo utilizes a method wherein motion tracing accuracy significantly outperforms (absolute) localization accuracy (80 cm level). Last but not least, the current version of the WiDeo implementation does not support localization in real-time.

The supermarket context, specifically tackled in this paper, is a typical example of a dynamic indoor space with dense obstacles and numerous moving objects (humans and shopping carts). In such a target context, real-time localization is of utmost importance for providing navigation service. Independent objects, e.g., shopping carts, not only have to be concurrently localized, but they also have to be unambiguously identified. For example, localization and identification should seamlessly work when target objects’ positions are very close (by means of both the estimated AoA and distance), or in cases wherein moving trajectories interfere and continuously overlap.

According to the abovementioned, the proposed Navindo indoor navigation system seems to be a suitable solution for a supermarket domain, given that it utilizes WSN topology and IR AoA sensors developed with such context in mind. The showcase application proved that the proposed localization solution can be easily deployed in order to provide accurate aisle level navigation in real-time.

The proposed IR-based localization principle is completely orthogonal to any RF-based solution, meaning that related approaches can be combined to boost localization performance for a given setup. The advantages of the proposed method are entirely complementary to the shortcomings of the RF localization systems.

## 5. Conclusions

In this paper, a supermarket navigation system, which relies on a novel IR AoA sensor, WSN-based localization infrastructure, and graph-based motion model, is introduced and described. The system is based on the LOS propagation of the IR signals, and a localization algorithm that uses measurements and AoA estimation provided by the IR AoA sensor. A proof-of-concept implementation demonstrated how inexpensive, autonomous, and easily deployable wireless nodes can be utilized to provide suitable localization accuracy for the target context. Several factors can have an impact on the localization error of the proposed solution, e.g., AoA measurement error, applied WSN topology (sensor density) for a given store layout, the relation between movement speed and IR transmit time delays, and movement trajectory (distance from the aisles graph). Altogether, four evaluation procedures were performed to investigate localization performance. The accuracy of estimated location was firstly observed in a 1D static context for different WSN densities, according to the given number of utilized AoA sensors and varied distance between WSN nodes and IR transmitters. The effect of moving speed on the localization accuracy in 1D and 2D setups was evaluated, as well, both empirically and via simulation. Since the proposed solution estimates cart location on the aisles graph exclusively, different movement trajectories were put under test: the straight 1D path along the sensor line, the straight 2D path beneath the sensor mesh, and the large-scale tortuous trajectory within the simulation environment. All obtained results, ranging from centimeter-level accuracy (Static-1D) to 1 m mean localization error (Mobile-2D simulation), are presented and discussed in detail.

Our future work plan consists of addressing detected limitations in the current version of the system and exploring potentials for further system improvement. This especially holds for a thorough investigation of the possibilities for increasing the IR signal range and analysis of the multipath propagation effects, as well as for evaluating the system in the real-world scenario, i.e., out of the laboratory context. As noted previously, we intend to upgrade the system platform by utilizing a state-of-the-art protocol suite with an enhanced hardware base that should support larger WSN topologies, as well as the implementation of power control through energy harvesting.

## Figures and Tables

**Table 1:** Taxonomy of the existing indoor localization methods.

**Infrastructure**	RF	WiFi, Bluetooth Low Energy (BLE), Ultra-wideband (UWB), Radio-frequency identification (RFID)
	Light	Visible Light Positioning (VLP), Infrared (IR)
**Infrastructure-free**		Magnetic, Sensor fusion, OCR

**Table 2:** Comparison of related indoor localization systems.

Method	Commercial Example	Typical Accuracy	Install. Costs	Energy Cons.	Main Drawbacks
VLP	ByteLight, Philips	50 cm	high	high	high computational requirements (real-time image processing) and installation costs
BLE	iBeacon (Apple)	>2 m	medium	low	low signal range, hard to achieve sub-meter precision
UWB	Sewio	30 cm	high	low	the need for precise time synchronization of anchor nodes, low range, specialized high-priced hardware design
WiFi	WiFiSLAM (Apple)	1–2 m	low	medium	site survey fingerprinting, high sensitivity to changes in the environment
Magnetic	IndoorAtlas	1–2 m	no	medium	magnetic field mapping, error increases with the size of the fingerprinting map
Sensor fusion	Project Tango	N/A	no	high	R&D phase, limited availability
IR AoA	proposed solution	3 cm (static 1D) 20–50 cm (mobile 1D) 40 cm (static 2D) <1 m (mobile 2D)	low	low	low range, IR collision

**Table 3:** Localization errors obtained in the simulation. Along with this paper, we provide a Supplementary Video File which thoroughly demonstrates error calculation in E4.

Cart Speed	Mean Error [cm]	STD [cm]
35 cm/s	63.4	39.8
70 cm/s	73.6	40.2
140 cm/s	99.5	50.5

## Supplementary Materials

The following are available online at https://www.mdpi.com/1424-8220/20/21/6278/s1, Video S1: E4-simulation.mp4.
